# The microbiota of farmed mink (*Neovison vison*) follows a successional development and is affected by early life antibiotic exposure

**DOI:** 10.1038/s41598-020-77417-z

**Published:** 2020-11-24

**Authors:** Martin Iain Bahl, Anabelle Legarth Honoré, Sanne Tygesen Skønager, Oliver Legarth Honoré, Tove Clausen, Lars Andresen, Anne Sofie Hammer

**Affiliations:** 1 National Food Institute, Technical University of Denmark, Kgs. Lyngby, Denmark; 2 Department of Veterinary Clinical and Animal Sciences, Faculty of Health and Medical Sciences, University of Copenhagen, Frederiksberg C, Denmark; 3 Danish Fur Breeders Research Centre, Holstebro, Denmark

**Keywords:** Antimicrobials, Bacteria, Microbial communities

## Abstract

On many mink farms, antibiotics are used extensively during the lactation period to reduce the prevalence and severity of pre-weaning diarrhoea (PWD) in mink kits (also referred to as greasy kit syndrome). Concerns have been raised, that routine treatment of PWD with antibiotics could affect the natural successional development of the gut microbiota, which may have long lasting consequences. Here we investigated the effects of early life antibiotic treatment administered for 1 week (postnatal days 13–20). Two routes of antibiotic administration were compared to a non-treated control group (CTR, n = 24). Routes of administration included indirect treatment, through the milk from dams receiving antibiotics by intramuscular administration (ABX_D, n = 24) and direct treatment by intramuscular administration to the kits (ABX_K, n = 24). A tendency for slightly increased weight at termination (Day 205) was observed in the ABX_K group. The gut microbiota composition was profiled by 16S rRNA gene sequencing at eight time points between Day 7 and Day 205. A clear successional development of the gut microbiota composition was observed and both treatment regimens caused detectable changes in the gut microbiota until at least eight days after treatment ceased. At termination, a significant positive correlation was identified between microbial diversity and animal weight.

## Introduction

The intestinal microbiota of mammals has attracted much attention during the last few decades, due to multitudes of interactions between the commensal bacteria and the human or animal host in both states of health and disease^1^. Colonization of the intestinal tract of human infants is initiated at birth, and generally follows a distinct successional pathway driven by mode-of-birth, lactation period, and first foods^2^. Evidence is strong concerning the importance of this natural successional development of the gut microbiota in relation to health including programming the immune system^3,4^ and weight regulation^5^. Early life exposure to antibiotics has a profound acute effect on the bacterial community composition^6^, and has been linked to negative health effects in later life, including changes in appetite regulation^7^. In mink production, it is common to use antibiotics before weaning to treat or reduce the risk of disease in the kits including gastro-intestinal disorders^8,9^. In 2018 the mink production in Denmark was 3.4 million females and the antibiotic consumption was 3700 kg of which 57% were beta-lactams^10^. Collectively the mink industry accounts for 4% of the total antibiotic consumption in animals in Denmark, despite representing only 2% of the live biomass^10^. Antibiotics are primarily administered to mink orally by mixing into water or feed, which may result in both healthy and sick animals being treated^9^. Diarrhoea and in particular pre-weaning diarrhoea (PWD) is the primary indication to initiate antibiotic treatment on mink farms. PWD (on the farms often referred to as “sticky kits”, “greasy kits” or “wet kits”) affects 1–4 week old mink kits and is characterized by watery diarrhoea and excessive secretion from apocrine glands, resulting in dark greasy exudation covering skin and nails^11,12^. The pathogenesis of PWD is assumed to be multifactorial. PWD may be initiated by entero-pathogenic viral agents and further complicated by secondary bacterial infections or intestinal dysbiosis. Astrovirus and calicivirus have been epidemiologically linked to PWD^13^. The primary concern related to use of antibiotics is the development, selection and potential spread of antibiotic resistant bacteria, which is suggested to be the reason for the observed increase of multi-resistant bacteria in the mink industry^14^. Another concern, is that antibiotic exposure in early life, may result in acute as well as long lasting disturbance of the gut microbiota affecting weight gain and future health^5^. The aim of this study was to profile the gut microbiota of mink during the growth season, and to investigate the effects of a single antibiotic treatment in early life, on the natural successional development of the gut microbiota and weight gain of the animals.

## Results

### Early life antibiotic exposure does not affect growth trajectories of mink kits

**Figure 1:**
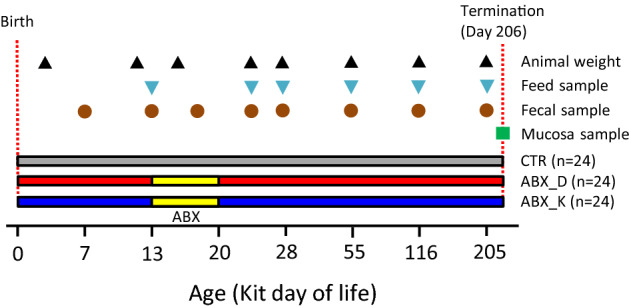
Experimental design. Coloured bars represent CTR (grey), ABX_D (red) and ABX_K (blue) groups with treatment period highlighted (yellow). Recording of weight and sampling of feed and intestinal contents was performed at the time-points indicated. Only animals that survived until Day 205 were included in the analysis.

**Figure 2:**
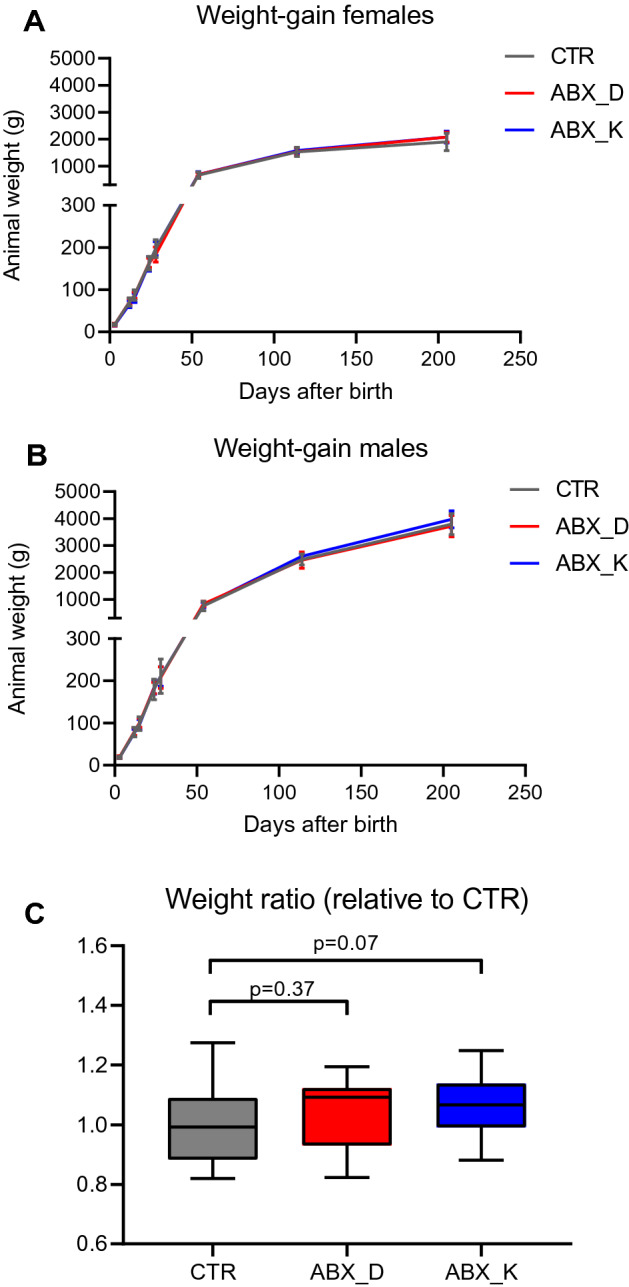
Animal weight gain. (**A**,**B**) The mean animal weight for female (**A**) and male (**B**) mink kits within each treatment group shown as a function of time (days after birth). Error bars indicate standard error of means. (**C**) Box-plot showing the weight ratio of animals in the ABX_D and ABX_K groups compared to the average weight of animals of the same sex in the CTR group on Day 205. The horizontal line shows the median value and whisker indicate total range. Differences between antibiotic treatment groups and the CTR group were assessed using Student’s t-test with p-values indicated.

Mink kits were divided into three groups (n = 24 per group) and followed for 205 days after birth, with antibiotic treatment occurring from Day 13–20, either by intramuscular injection in the lactating females (ABX_D) or the kits directly (ABX_K) compared to non-treated controls (CTR) (Fig. 1 and Table 1). Treatment with antibiotics during early life did not affect the overall weight gain of female or male kits (Fig. 2A,B), although a slight tendency for increased relative weight-gain in the ABX_K group (compared to the CTR group) was observed when sexes were combined (p = 0.07, Student’s *t*-test) (Fig. 2C). During the study, 10 of the 72 kits died unexpectedly. It was not possible to identify the cause of death, which did not appear to be related to the different treatments, but was dependent on litter. On Day 28, mink kits displaying characteristic symptoms of PWD were found in two of four litters in the ABX_D group and one of four litters in the ABX_K group, with no litters affected in the CTR group (0/4). In the affected litters, all 6 kits displayed symptoms of PWD preventing statistical conclusions to be made on the potential negative effects of antibiotic exposure.

**Table 1:** Baseline data for mink kits.

	CTR	ABX_D	ABX_K
Number of litters	4	4	4
Litter size	6	6	6
Number of males	12	13	14
Average weight (g)	17.6	18.2	17.6
Average dam age (years)	1.5	1.5	1.5

*CTR* control group, *ABX_D* antibiotic treatment of lactating females through intramuscular injection, *ABX_K* mink kits treated with antibiotics directly through intramuscular injection.

### The gut microbiota of mink kits follows a successional development

**Figure 3:**
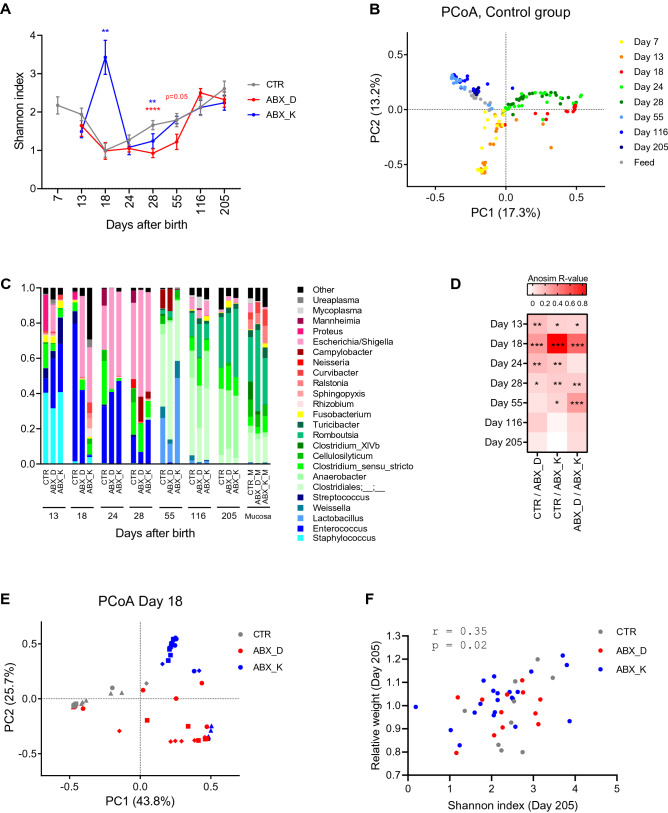
Temporal development of microbiota in mink kits and effect of early life antibiotic exposure. (**A**) Alpha diversity shown as Mean Shannon index of faecal samples collected at different time point from animals in the CTR, ABX_D, ABX_K groups. Error bars indicate standard error of means. Differences between antibiotic treatment groups and the CTR group were assessed using Mann–Whitney test with p-values indicated. (**B**) Beta diversity shown as Principle coordinate analysis (PCoA) based on Bray–Curtis distances of faecal samples collected at different time points from the CTR group as well as feed samples. (**C**) Bacterial composition in mink kits from the CTR, ABX_D, ABX_K groups at different time points shown as average relative abundance at the genus level. Bacterial genera representing less than 4% on average in any of the groups were aggregated into one category (Other). The different genera are coloured in grades of Blue, Bacilli; Green, Clostridia; Yellow, Fusobacterium, Red, Proteobacteria; Gray, Mollicutes. (**D**) Heatmap illustrating analysis of similarities (ANOSIM) between indicated groups at the different time points. (**E**) Principle coordinate analysis (PCoA) based on Bray–Curtis distances of faecal samples collected from groups ABX_D, ABX_K and CTR on Day 18, during the antibiotic treatment period. Colours show groups and different symbols are used to indicate litter. (**F**) Spearman’s Rank correlation analysis between microbial Shannon index and relative animal weight on Day 205. In panels (**A**) and (**D**): *p < 0.05; **p < 0.01; ***p < 0.001; ****p < 0.0001.

In the non-treated kits (CTR) we observed an initial decrease in alpha diversity (Shannon index) from Day 7 to Day 18 after birth (p = 0.0003, Student’s *t*-test) , which was followed by a sustained increasing trajectory of mean values until termination on Day 205 (Pearson r = 0.93, p = 0.0077) (Fig. 3A). During the same period, a successional development of the gut microbiota was observed (Fig. 3B,C), with a clear shift in microbiota composition in the CTR group between Days 13 and 18 (R = 0.78, p < 0.001, ANOSIM). This change was characterized by a marked reduction in the relative abundance of *Staphylococcus* spp. (p < 0.0001, Mann–Whitney test) and an increase in *Enterococcus* spp. (p = 0.002, Mann–Whitney test) (Fig. 3C). Between Days 28 and 55, another marked shift was observed (R = 0.97, p < 0.001, ANOSIM) driven by a decrease in *Enterococcus* spp., *Escherichia* spp. and *Clostridium *sensu stricto and an increase in relative abundance of *Lactobacillus* spp., *Weissella* spp. and unclassified *Clostridiales* (Fig. 3C). In samples from mucosal scrapes in the CTR group, we found increased relative abundance of the genera *Ralstonia*, *Curvibacter*, *Burkholderia*, *Pelomonas*, *Frederiksenia* and *Clostridium_XIVb* and decreased relative abundance of genera *Anaerobacter*, *Clostridium_XI* and *Terrisporobacter* compared to faecal samples at termination (ANCOM analysis).

### The faecal microbiota of mink is characterized by large individual variation

We observed a high degree of variation in bacterial community composition between animals at the different sampling time-points (Fig. S1). This variation was to some extent caused by a litter effect, which was most pronounced for kits in litter 4 (green), which during the first 2 weeks of life were vastly dominated by *Proteus* spp., representing up to 95.4% of the community, which then gradually decreased in relative abundance. Litter 2 (yellow) was found to be enriched for *Mannheimia* spp. between days 18 and 28 compared to the other litters (Fig. S1).

### Mink Feed samples contain a high diversity of bacteria dissimilar to the gut microbiota

Analysis of the bacterial composition of mink feed revealed time-dependent variation. On sampling days 13, 28 and 55 the bacterial community of the mink feed samples was vastly dominated by *Lactobacillus* spp. and *Weisella* spp., whereas on days 24, 116 and 205 a high diversity of bacteria were found (Fig. S2A). Overall, there were large compositional differences in microbiota between feed samples and faecal samples at all sampling time-points (Fig S2A,B).

### Antibiotic treatment has both an acute and long term effect on the gut microbiota

Treatment with Amoxicillin (AMX) caused changes in the microbial diversity in both kits of dams treated with the antibiotic (ABX_D) and kits treated directly (ABX_K) compared to the control group (Fig. 3A,C,D). The most pronounced effect was observed in the ABX_K group with a surge in Shannon diversity index, compared to the CTR group, on Day 18 (p = 0.0021, Mann–Whitney test), which is during the antibiotic administration period (Day 13–20). After the administration of antibiotics ceased, both treatment groups had lower diversity on Day 28 compared to controls, but no difference between groups was observed from Day 116 until termination (Fig. 3A). Antibiotic treatment also affected the bacterial composition on Day 18 dependent on administration route (Fig. 3C,E). Analysis of compositional differences (ANCOM test) on Day 18, revealed a higher number of compositional changes in the kits administered directly (ABX_K) compared to controls (CTR group) than in kits of dams treated with amoxicillin (ABX_D) compared to CTR, with 23 and 4 differently abundant genera respectively (Supplementary Table S1). In the ABX_K group there was a significant decrease in relative abundance of *Enterococcus* and *Vagococcus*, while 21 other genera increased. In the ABX_D group, *Streptococcus*, *Stapylococcus* and *Chryseobacterium* decreased, while *Escherichia* was shown to increase (ANCOM test). Comparison between the two antibiotic treated groups on Day 18 revealed 15 differently abundant genera (Supplementary Table S1). Differences in bacterial composition gradually decreased and from Day 116 onwards, no differences between antibiotic treated animals and controls were observed based on Bray–Curtis distances (p > 0.05, ANOSIM) (Fig. 3D) and ANCOM tests.

### Microbial diversity is linked to weight gain

We investigated links between the intestinal microbial diversity and weight of the animals at termination. A weak but significant correlation (rho = 0.35, p = 0.02, Spearman correlation) was found between the Shannon index of diversity on Day 205 and the relative animal weight (compared to the CTR group) on the same day (Fig. 3F). We investigated whether microbial diversity in early life could predict animal weight at termination, but found no significant correlations at any of the other time-points.

## Discussion

In the present study we profiled the successional development of the mink microbiota from birth until pelting and investigated acute and long term effects of antibiotic treatment, during early life.

To our knowledge, this represents the first long-term assessment of intestinal microbiota development in mink. Sequencing of faecal samples from the non-treated animals, revealed a successional development of the microbiota, during the entire study period of 205 days, which has characteristics that are dissimilar to that of humans. For the first weeks of life, during the lactation period, the mink kits were predominately colonized by *Staphylococcus* spp. with a significant shift observed on Day 18 towards dominance of *Enterococcus* spp. Since several *Staphylococcus* spp. are considered pathogens of mink^15^ and have been linked to diarrhoea in juvenile mink and ferrets^16,17^, the identification of natural *Staphylococcus* spp. in pre-weaning mink may be misinterpreted as primary or secondary intestinal infection and lead to antimicrobial treatment. Interpretation of *Staphylococcal* spp. in cultures from the intestine of sick mink should be made with caution, since the results of this study indicates that a microbiota dominated by *Staphylococcus* spp. may represent a microbial development step also found in healthy mink kits. A more complete understanding of the development of the mink microbiota represents an important basis for understanding the role of intestinal microbes in health and disease of the mink and also for the optimization of prevention and treatment protocols.

Contrary to the early life microbiota of humans, we observed almost no species belonging to *Bifidobacteria* spp. and *Bacteroides* spp. which are selected for by human milk oligosaccharides^18^. The observed dominance of *Enterococcus* spp. could in part reflect environmental conditions, allowing growth of this facultative anaerobic bacterial genera. A more anaerobic gut environment could explain the later dominance of bacteria belonging to the Clostridia class from Day 55, which could be increased further with feed-borne species^19^. Danish farm mink are fed with a commercially produced wet feed, consisting of a mix of animal by-products and slaughter offal e.g. by-products from the fishing and meat industries and plant origin such as corn gluten meal, soybean oil and extruded cereals^20^. There may be high bacterial counts in some by-products used in mink feed, including fish by-products, slaughterhouse by-products and unpreserved slaughter blood^21^. The ready-to-eat feed may contain harmless bacterial species including some types of clostridia^20^, which may contribute to the intestinal microbiota, as well as potentially pathogenic or toxigenic bacteria, originating from contaminated raw materials^22,23^.

Mink dams or kits were treated with amoxicillin by intramuscular injection, as opposed to oral administration through feed or water, to ensure correct dosing. Amoxicillin is known to be excreted through the biliary route in humans^24^ and thus enter the intestinal lumen potentially causing effects on the resident bacterial community. The present study confirms that amoxicillin also entered the intestinal tract in mink and caused acute changes in bacterial community diversity and composition (Fig. 3C–E). Interestingly, we also found significant acute shifts in bacterial profiles in mink kits exposed only indirectly through the dam, which is very likely due to amoxicillin passing into the milk compartment. Transfer of amoxicillin to suckling mink following both oral as well as intra-muscular treatment of the dams, has recently been demonstrated, showing that peak concentrations in the serum of kits occurred eight hours after administration and was not affected by the route of administration^25^. A significant difference in the microbial response to the two exposure routes was found (Fig. 3E), which is likely caused by differences in concentrations of the drug in the intestine of the kits^26^. In both treatment groups we observed a surge in *Escherichia* spp. (Fig. 3C), which is consistent with previous observations in rats^7,27^, dogs^28^ and pigs^29^, despite this group of bacteria being generally regarded as susceptible. The presence of *Enterobacteriaceae* in early colonization is normal also in humans^30^ but can also result in disease^27,31^. The observed acute and dramatic increase in Shannon diversity in the ABX_K group on Day 18 during antibiotic treatment contradicts studies in other animal species, generally displaying reduction in Shannon diversity during antibiotic treatment^32,33^, but is consistent with a previous study in mink^34^, and probably reflects the very low bacterial load in mink compared to other species. We did not see any change in Shannon diversity in the ABX_D group on Day 18 (Fig. 3A), which is consistent with the observation of fewer differentially abundant species in this group compared to the kits administered directly with antibiotics (4 versus 23 genera) and is most probably caused by the in-direct antibiotic administration in this group.

Reports of antimicrobial use in mink reveal, that the oral administration route constitutes 98% of the antimicrobial use measured in defined animal daily dose. The antimicrobial use has a clear seasonal pattern, with high treatment proportions in the month of May, due to treatment of the pre-weaning mink^9^. Oral administration of antimicrobials in mink during the lactation period often includes either part of, or the entire farm, including both sick and asymptomatic animals.

In this study symptoms of PWD developed in both treatment groups, but not in the control group. Epidemiological investigations have indicated that PWD has a primary viral aetiology^12,13^, which may explain that antimicrobial treatment does not prevent PWD symptoms in the treatment groups. Studies in mice and rats have indicated that the impact of antimicrobial treatment on the intestinal microbiota may render the organism more susceptible to some infectious agents, including both bacteria and virus^35,36^. In the affected litters in this study all 6 kits displayed symptoms of PWD preventing statistical conclusions to be made on the potential negative effects of antibiotic exposure. Finally, we observed a weak but significant positive correlation between Shannon diversity index and body weight at the time of pelting (Fig. 3F). This is interesting as it suggests that the microbiota composition may affect the growth rate of mink, which has not been recognized previously.

## Materials and methods

### Ethics statement

All institutional and national guidelines for the care and use of laboratory animals were followed. The handling of the animals and the experimental procedures were approved by the Danish Animal Experiments Inspectorate (licence no. 2016-15-0201-00965) and all personnel involved in handling and care of the animals were trained for carrying out animal experiments. The study was carried out according to legislation of the Danish Medicines Agency.

### Animals and housing

The study was carried out at the Danish Fur Breeders Association research farm, Kopenhagen Fur, Holstebro, Denmark. All animals had received standard vaccinations (distemper virus vaccine, Distemink Vet., Biovet ApS and combined virus enteritis, botulism and pseudomonas vaccine Biocom-P Vet., Biovet ApS) at the age of 10 weeks and the farm was tested negative for Aleutian disease virus. The mink dams were housed individually in standard-sized cages including a nesting box and bedding, in 2-rowed open sheds. The mink were fed once a day in the morning with wet feed consisting of a mix of animal by-products and slaughter offal produced by a local feed production facility. The mink had free access to water. Experienced animal caretakers were responsible for the routine handling and care of the animals. Antibiotics were administered by veterinarians or veterinary master students.

### Experimental design

Twelve litters were randomly selected among litters containing six kits, born on the 29th of April 2017 and of brown colour type. All mink kits were identified individually by placement of a microchip (Uno Pico transponder, UNO BV, PC Zevenaar, Holland) in the back of the neck. The 12 litters were randomly allocated to one of two different antibiotic treatment regimens (n = 24 kits per group) or a control group (CTR, n = 24 kits) not receiving antibiotics (Fig. 1, Table 1). The antibiotic used in this study was Curamox Prolongatum Vet, amoxicillinum trihydricum, 150 mg/ml. In the ABX_D group, only the adult lactating females were treated with 75 mg amoxicillin (0.5 ml) by intramuscular injection (IM). In the ABX_K group, only mink kits (not their mothers) were treated with 15 mg amoxicillin (0.1 ml) IM. There are no antimicrobial products registered for mink in Denmark, so there are no approved dosages available. Amoxicillin was used because this drug is often applied for the treatment of PWD in Danish mink farms^37^. The dosages used in this experiment were based on personal experience from mink veterinarians. Moreover the dosage used corresponded to the recommended dosage range for dogs and cats (10–15 mg per kg live body weight) following local guidelines. The antibiotics were administered IM in order to control the dosage given to each animal and avoid any potential effect of individual variation in intake of medicated food and water. The antibiotic treatment was given every 48 h over the course of 8 days (4 times in total) between days 13 and 19 *post partum* (*pp*). Stool samples were collected from all kits individually on Days 13, 18, 24, 28, 55, 116 and 205 pp as well as from the CTR group on Day 7 (Fig. 1). Defecation was stimulated by gently brushing sterile cotton swabs in the perianal area and stool was collected on the sterile cotton swabs, distributed in to cryo-vials and placed on dry ice until storage at − 80 °C. Feed samples were also collected at most of the same time points during the study and treated similarly (Fig. 1). During the study period the growth of kits were monitored and any clinical symptoms in the animals were recorded by the farm veterinarian. In order to investigate effects of antibiotics on weight gain at termination (Day 205), the relative animal weights were calculated, by dividing the individual animal weight with the average weight in the CTR group for animals of the same sex. Characteristic symptoms of PWD were classified as watery diarrhoea, greasy exudation on the nails (referred to as black nails) and greasy exudation on the fur. A clinical diagnosis of PWD was made when all these symptoms were present in the mink kit. Kits were euthanized and submitted to routine *post mortem* examination performed by a veterinarian following the end of the study period. Mucosal scrapes were taken from the large colon of all animals at termination for microbiota analysis as previously described^34^.

### Microbial profiling

Total bacterial DNA was extracted from all faecal and feed samples (approximately 100 mg) using the MoBio Power Soil-htp 96 Well Soil DNA Isolation Kit (MoBio Laboratories, Carlsbad, CA, USA) according to the manufacturer’s recommendations, with minor modifications^38^. DNA concentrations were determined using the Quant-iT dsDNA HS kit (LifeTechnologies). A total of nine 16S rRNA gene libraries, collectively containing all samples, were prepared by PCR amplification of the V3-region as previously described with up to 90 samples represented in each library^39^. Partial 16S rRNA gene sequencing was performed on an Ion Torrent Personal Genome Machine (PGM™, ThermoFisher Scientific) using Ion PGM Hi-Q kit, 200 bp sequencing and Ion 318™ Chip. The CLC Genomic Bench software version 12 (Qiagen) was used to de-multiplex samples and trim reads for PCR primers and adaptors after which samples were exported as individual FASTQ files. Next, the DADA2 pipeline (version 1.12.1)^40^ implemented in RStudio^41^ was used to generate an amplicon sequence variant (ASV) table (MaxEE = 1, pool = TRUE) and taxonomic classification of the inferred ASVs based on the Ribosomal Database Project (rdp_train_set_16)^42^. The QIIME2 pipeline^43^ was used for downstream processing of the ASV table. A total of 619 samples were included in the study with a median number of high quality reads of 38,820 (13–177,959) which represented a total of 1,223 features (ASVs). Samples with less than 10,000 reads were discarded (34 samples) and the *qiime diversity core-metrics-phylogenetic* script with a sampling depth of 10,000 was run to determine alpha- and beta diversity as well as taxonomical composition in the 585 samples. Subsets of samples were also used as input for this script to generate principle coordinate analysis on specific days. Taxonomy distribution plots were prepared using aggregated relative abundances at the genus level with any genera representing less than 4% of the total community in any day/treatment group assigned to the category "Other".

### Statistics

Statistical tests were performed in GraphPad Prism version 8 unless otherwise indicated. The two-sample Students t-test was used unless variances were different between groups, in which case the non-parametric Mann–Whitney test was used. Associations between Shannon diversity index and animal weight was investigated by calculating Spearman Ranks correlations. To determine differences in overall bacterial community structure the Analysis of similarities comparisons between groups (ANOSIM) test was used (incorporated in QIIME2), with a Bray–Curtis distance matrix as input. The Analysis of composition of microbiomes (ANCOM) test (incorporated in QIIME2) was used to investigate differentially abundant taxa between groups.

## Supplementary information

Supplementary Information

## Data Availability

The 16S rRNA gene sequence data are deposited in the NCBI Sequence Read Archive with the accession number PRJNA630170.
