# Supplementary material for: The microbiota of farmed mink (Neovison vison) follows a successional development and is affected by early life antibiotic exposure
Source: Sci Rep. 2020 Nov 24;10:20434. doi: 10.1038/s41598-020-77417-z (PMC7686315; doi:10.1038/s41598-020-77417-z)
Supplement: Supplementary file 1 — Supplementary Information [file 41598_2020_77417_MOESM1_ESM.pdf]

The microbiota of farmed mink (*Neovison vison*) follows a successional development and is affected by early life antibiotic exposure

Martin Iain Bahl[1*], Anabelle Jakobsen[2], Sanne Tygesen Skønager[2], Oliver Lykke Honoré[2], Tove Clausen[3], Lars Andresen[2], Anne Sofie Hammer[2]

[1]National Food Institute, Technical University of Denmark, Kgs. Lyngby, Denmark

[2]Department of Veterinary Clinical and Animal Sciences, Faculty of Health and Medical Sciences, University of Copenhagen, Frederiksberg C, Denmark

[3]Danish Fur Breeders Research Centre, Holstebro, Denmark

SUPPLEMENTARY INFORMATION

Table of contents

Supplementary Figure S1 2
Supplementary Figure S2 3
Supplementary Table S1 4

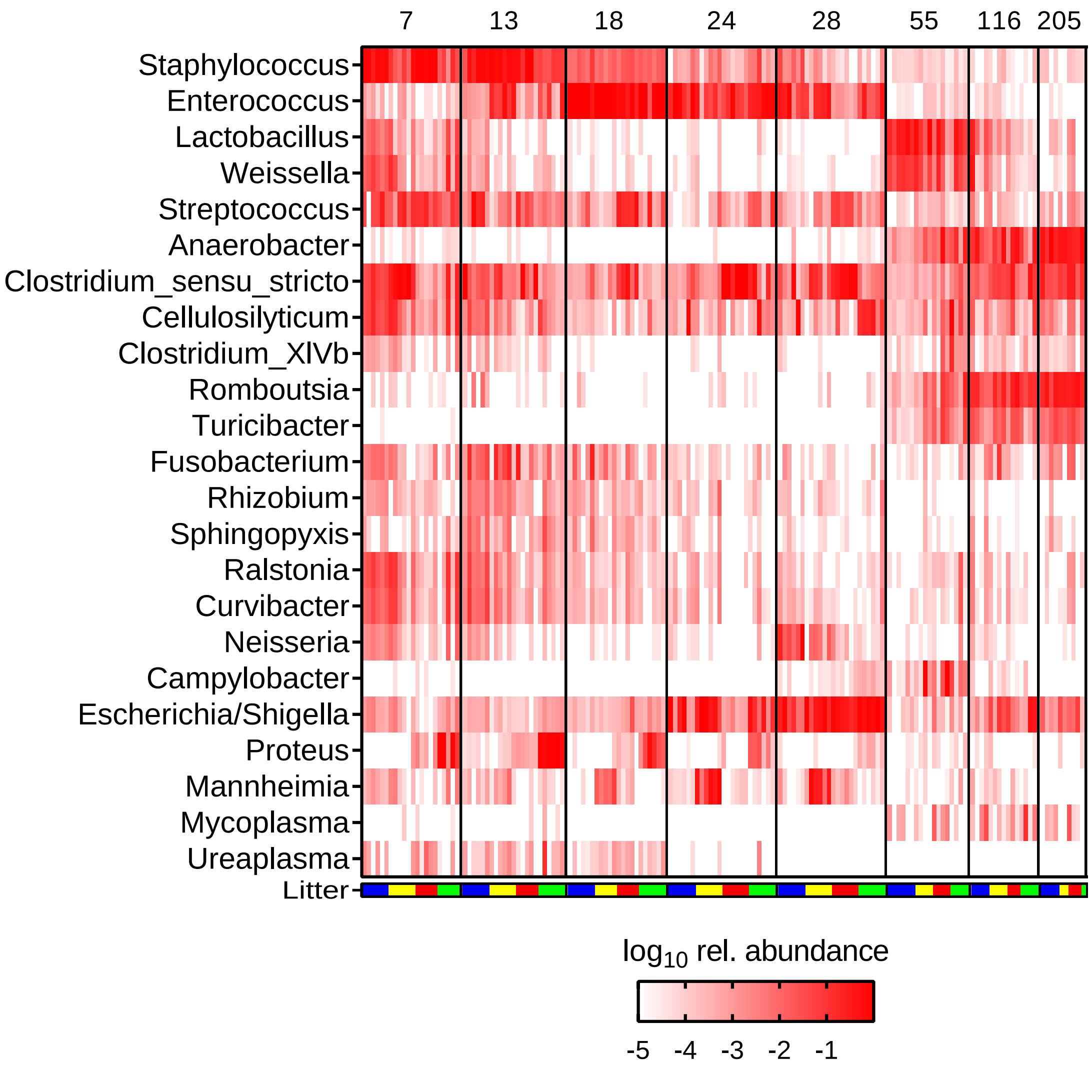

Figure S1. Heatmap of relative abundances at the genera level for the CTR group. Each column represents a single animal with litter indicated by different colours at the bottom; litter 1: blue, litter 2: yellow, litter 3: red and litter 4: green. Sampling time-points (days) are shown above the heatmap.

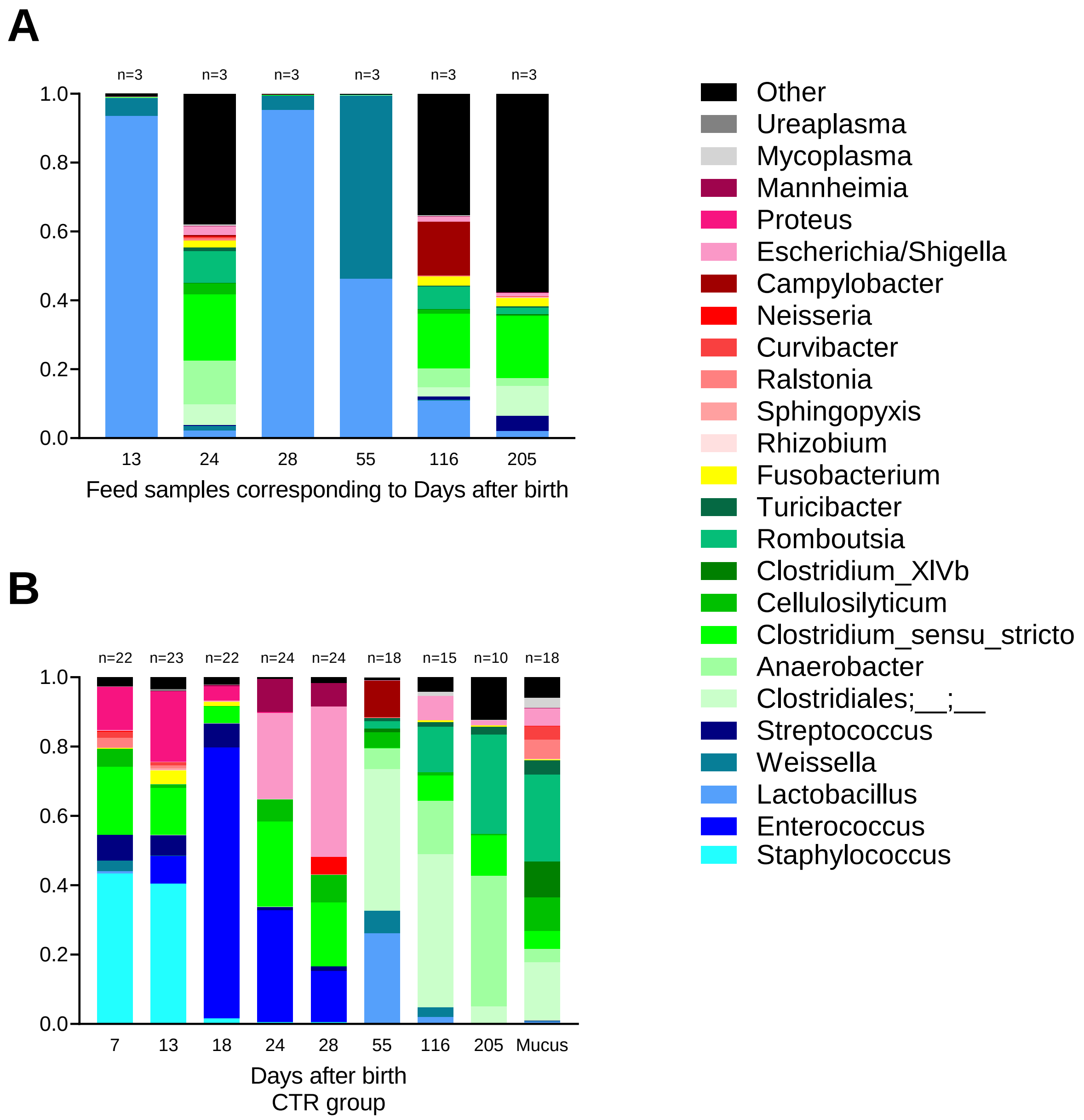

Figure S2. Bacterial composition in samples from feed and faecal samples. (A-B) Bacterial composition in feed sample (A) and faecal samples from animals in the CTR group (B) at different time points shown as average relative abundance at the genus level. Bacterial genera representing less than 4% on average in any of the groups were aggregated into one category (Other). The different genera are coloured in grades of Blue, Bacilli; Green, Clostridia; Yellow, Fusobacterium, Red, Proteobacteria; Gray, Mollicutes.

Supplementary Table S1. Differentially abundant genera on Day 18 between groups (ANCOM analysis).

Genera	ABX_K / CTR	ABX_D / CTR	ABX_K /ABX_D
Bacteria;__Actinobacteria;__Actinobacteria;__Actinomycetales;__Microbacteriaceae;__Agrococcus	↑		
Bacteria;__Actinobacteria;__Actinobacteria;__Actinomycetales;__Microbacteriaceae;__Clavibacter	↑		↑
Bacteria;__Actinobacteria;__Actinobacteria;__Actinomycetales;__Microbacteriaceae;__Leifsonia	↑		↑
Bacteria;__Actinobacteria;__Actinobacteria;__Actinomycetales;__Microbacteriaceae;__Microbacterium	↑		↑
Bacteria;__Actinobacteria;__Actinobacteria;__Actinomycetales;__Microbacteriaceae;__Rathayibacter	↑		
Bacteria;__Bacteroidetes;__Flavobacteriia;__Flavobacteriales;__Flavobacteriaceae;__Chryseobacterium		↓	
Bacteria;__Bacteroidetes;__Sphingobacteriia;__Sphingobacteriales;__Sphingobacteriaceae;__Pedobacter	↑		
Bacteria;__Firmicutes;__Bacilli;__Bacillales;__Staphylococcaceae;__Staphylococcus		↓	
Bacteria;__Firmicutes;__Bacilli;__Lactobacillales;__Enterococcaceae;__Enterococcus	↓		↑
Bacteria;__Firmicutes;__Bacilli;__Lactobacillales;__Enterococcaceae;__Vagococcus	↓		↓
Bacteria;__Firmicutes;__Bacilli;__Lactobacillales;__Leuconostocaceae;__Weissella	↑		
Bacteria;__Firmicutes;__Bacilli;__Lactobacillales;__Streptococcaceae;__Streptococcus		↓	
Bacteria;__Firmicutes;__Clostridia;__Clostridiales;__Lachnospiraceae;__Cellulosilyticum	↑		↑
Bacteria;__Proteobacteria;__Alphaproteobacteria;__Caulobacterales;__Caulobacteraceae;__Caulobacter	↑		
Bacteria;__Proteobacteria;__Alphaproteobacteria;__Rhizobiales;__Aurantimonadaceae;__Aureimonas	↑		↑
Bacteria;__Proteobacteria;__Alphaproteobacteria;__Rhizobiales;__Hyphomicrobiaceae;__Devosia	↑		↑
Bacteria;__Proteobacteria;__Alphaproteobacteria;__Rhizobiales;__Methylobacteriaceae;__Methylobacterium	↑		↑
Bacteria;__Proteobacteria;__Alphaproteobacteria;__Rhizobiales;__Rhizobiaceae;__Ensifer	↑		
Bacteria;__Proteobacteria;__Alphaproteobacteria;__Rhizobiales;__Rhizobiaceae;__Rhizobium	↑		↑
Bacteria;__Proteobacteria;__Alphaproteobacteria;__Sphingomonadales;__Sphingomonadaceae;__Sphingomonas	↑		
Bacteria;__Proteobacteria;__Alphaproteobacteria;__Sphingomonadales;__Sphingomonadaceae;__Sphingopyxis	↑		↑
Bacteria;__Proteobacteria;__Betaproteobacteria;__Burkholderiales;__Burkholderiaceae;__Ralstonia	↑		↑
Bacteria;__Proteobacteria;__Betaproteobacteria;__Burkholderiales;__Comamonadaceae;__Curvibacter	↑		↑
Bacteria;__Proteobacteria;__Betaproteobacteria;__Burkholderiales;__Comamonadaceae;__Variovorax	↑		↑
Bacteria;__Proteobacteria;__Gammaproteobacteria;__Enterobacteriales;__Enterobacteriaceae;__Escherichia/Shigella		↑	
Bacteria;__Proteobacteria;__Gammaproteobacteria;__Pseudomonadales;__Pseudomonadaceae;__Pseudomonas	↑		
Bacteria;__Proteobacteria;__Gammaproteobacteria;__Xanthomonadales;__Xanthomonadaceae;__Stenotrophomonas	↑		↑
Number of differentially abundant genera	23	4	15
